# Racial Disparities in Malignant Primary Brain Tumor Survival in Texas From 1995 to 2013

**DOI:** 10.7759/cureus.11710

**Published:** 2020-11-25

**Authors:** Solomon Ambe, Kristopher A Lyon, Janice Oh, M. Karen Newell Rogers, Olalekan Olanipekun, Nduma N Basil, Ekokobe Fonkem

**Affiliations:** 1 Neurology, Tulane University School of Medicine, New Orleans, USA; 2 Neurosurgery, Baylor Scott & White Medical Center - Temple, Temple, USA; 3 Internal Medicine, Cedars-Sinai Medical Center, Los Angeles, USA; 4 Neurology, BCell Solutions, Inc, Colorado Springs, USA; 5 Neurology, Advocate Illinois Masonic Medical Center, Chicago, USA; 6 Neurology, Ahmadu Bello University, Zaria, NGA; 7 Neurology, Barrow Neurological Institute, Phoenix, USA

**Keywords:** brain tumor, epidemiology, geography, race, socioeconomic class, poverty index

## Abstract

Background: Differences among the top five races in Texas will be explored to determine if racial, geographic, and healthcare disparities exist in patients undergoing treatment for a primary malignant brain tumor.

Methods: Data were obtained from the Texas Cancer Registry from 1995 to 2013. SAS 9.3 (SAS Institute, Inc., Cary, NC) and SEER*Stat 8.3.2 (National Cancer Institute, Bethesda, MD) software were used to analyze death from malignant brain tumors and cause-specific survival. Survival rates were compared using Kaplan-Meier curves and Log-Rank tests. Hazard ratios were estimated using the Cox proportional hazards regression model.

Results: Median survival was highest among Asians at 92 months (95% CI: 72, 142) and least among Whites at 20 months (95% CI: 19, 21). Patients living in the Upper Gulf Coast region of Texas had the longest survival time at 31 months (95% CI 29-35%), while those patients in the Texas Panhandle had the shortest survival time at 18 months (95% CI 14-23%). Patients with a poverty index of 0-5% had the highest median survival time of 32 months (95% CI 29-35%), as compared to patients with a poverty index of 10-20% who had a median survival of 22 months (95% CI 21-24%).

Conclusions: Ethnic minorities and higher socioeconomic class demonstrated survival advantage. White males had the worst survival of those with primary malignant brain tumors. Other significant factors affecting a patient’s survival rate included geographic location, poverty index, sex, and age, thus suggesting a potential genetic and environmental influence.

## Introduction

In general, primary malignant brain tumors result in a poor prognosis for the patient, with a relatively low 33.7% five-year relative survival rate [1]. Although many factors may contribute to a patient’s prognosis after diagnosis with a primary brain tumor, this study will analyze selected social determinants of health. Specifically, identifying the underlying differences among ethnic groups, their associated access to healthcare, and the environment in which patients live may influence the outcome of patients undergoing treatment for a primary malignant brain tumor. Historically, minority groups were considered to have a lower socioeconomic status with reduced access to a healthcare provider, surgeon, and postoperative adjuvant therapy [2]. This consequently was thought of as leading to a decreased survival rate among minorities with primary brain tumors. Unfortunately, the literature remains inconsistent regarding what contribution a patient’s race may have on their prognosis after diagnosis with a malignant primary brain tumor. For example, one large study found that African Americans had a 13% increased risk of death from primary malignant brain cancers when compared to other ethnicities, while a different study revealed improved survival of African American brain tumor patients when compared to other ethnicities [2,3].

Investigating the survival rates of insured patients with malignant brain tumors with ample access to healthcare versus the uninsured patient likewise have revealed mixed data. One study showed that the insured patient who had a prior established relationship with a primary care provider would have on average twice as long as lifespan after a diagnosis with a brain tumor compared to an uninsured patient [3,4]. However, a retrospective study demonstrated no relationship between survival and socioeconomic status as determined by household income [5]. Another social determinant of survival in brain cancer is the geographic location in which the patient lives. Several studies have discovered significant differences in survival of primary brain cancer by geographical regions of various sizes, including citizens in a multicity metropolis, citizens in the same state, and citizens in a multistate region of the United States [6].

Given the population size, racial diversity, inequality to healthcare, and geographic variety, citizens of the state of Texas will be studied as a representative sample of the United States’ population. In this study, the significant predictors leading to an increased rate of death after diagnosis with a malignant primary brain tumor in Texas from 1995 to 2013 will be determined. In addition, the variables of sex, race, geographical region, and poverty level will be analyzed to assess their significance in regards to survival rate after diagnosis with a primary malignant brain tumor. By identifying the variables within one large statewide dataset, disparities in healthcare may be identified and addressed to improve the survival of patients with primary brain cancers.

## Materials and methods

Data were obtained from the Texas Cancer Registry, a statewide population-based registry that serves as the foundation of measuring cancer burden in Texas and one of the largest cancer registries in the United States [7]. The study population included patients with a primary malignant brain tumor diagnosed in Texas between the years of 1995 and 2013. The data were obtained from hospitals, cancer treatment centers, ambulatory surgical centers, and pathology laboratories located across the state. Given the retrospective nature of this study involving de-identified patient information, an IRB was not required by our institution. The object of interest was primary malignant brain tumor patients based on the International Classification of Diseases for Oncology, 3rd Edition, as defined by the World Health Organization. Malignant behavior was defined by the surveillance epidemiology and end result (SEER) program of the National Cancer Institute with each tumor’s primary location consistent with the Central Brain Tumor Registry of the United States (CBTRUS). The locations studied were the cerebral meninges (C70.0), meninges not specified (C70.9), cerebrum (C71.0), frontal lobe (C71.1), temporal lobe (C71.2), parietal lobe (C71.3), occipital lobe (C71.4), ventricle (C71.5), cerebellum (C71.6), brain stem (C71.7), overlapping lesion of the brain (C71.8), brain not specified (C71.9), olfactory nerve (C72.2), optic nerve (C72.3), acoustic nerve (C72.4), cranial nerve not specified (C72.5), overlapping lesion of brain and CNS (C72.8), nervous system not specified (C72.9), pituitary gland (C75.1), craniopharyngeal duct (C75.2), and pineal gland (C75.3).

Kaplan Meier survival curve with the Log-Rank test was used to compare the survival rates across the racial groups, poverty index, and geographical location. The event of interest, death from primary malignant brain tumors, was analyzed using cause-specific survival. The Cox regression proportional hazard model estimated the hazard ratios. Geographic location was grouped into seven different regions of Texas based on distinct cultures and geography [8]. The median survival time based on a patient’s sex, race, geographical location, and poverty index was estimated with a 95% confidence interval. SAS version 9.3 (SAS Institute, Inc., Cary, NC), and SEER*Stat 8.3.2 (National Cancer Institute, Bethesda, MD) software was used for the analysis.

## Results

A total of 26,070 people met the inclusion criteria for the study. The mean age of the studied population was 48.4±23.8 years. Males constituted 55.1% of the population, and females constituted 44.9%. Five major races were represented to include white non-Hispanics (66.7%), Hispanics (22.9%), Blacks (7.6%), Asian/Pacific Islander (1.8%), and American Indian/Alaska Natives (0.2%). Malignant primary brain tumors with the highest frequency that met our inclusion criteria included glioblastoma (36.8%), astrocytoma (16.5%), glioma (8.7%), oligodendroglioma (5.5%), diffuse large B-cell non-Hodgkin lymphoma (3.6%), and ependymoma (0.6%). The Cox regression proportional hazard model revealed that the geographical region, poverty index, race, and gender were significant predictors in the hazard of death from a primary brain tumor. Of note, younger age at diagnosis also had a protective effect, as each increase in age by one year at the time of diagnosis increased the risk of death by 3.5% (p-value < 0.0001).

Survival by geographic region

**Figure 1:**
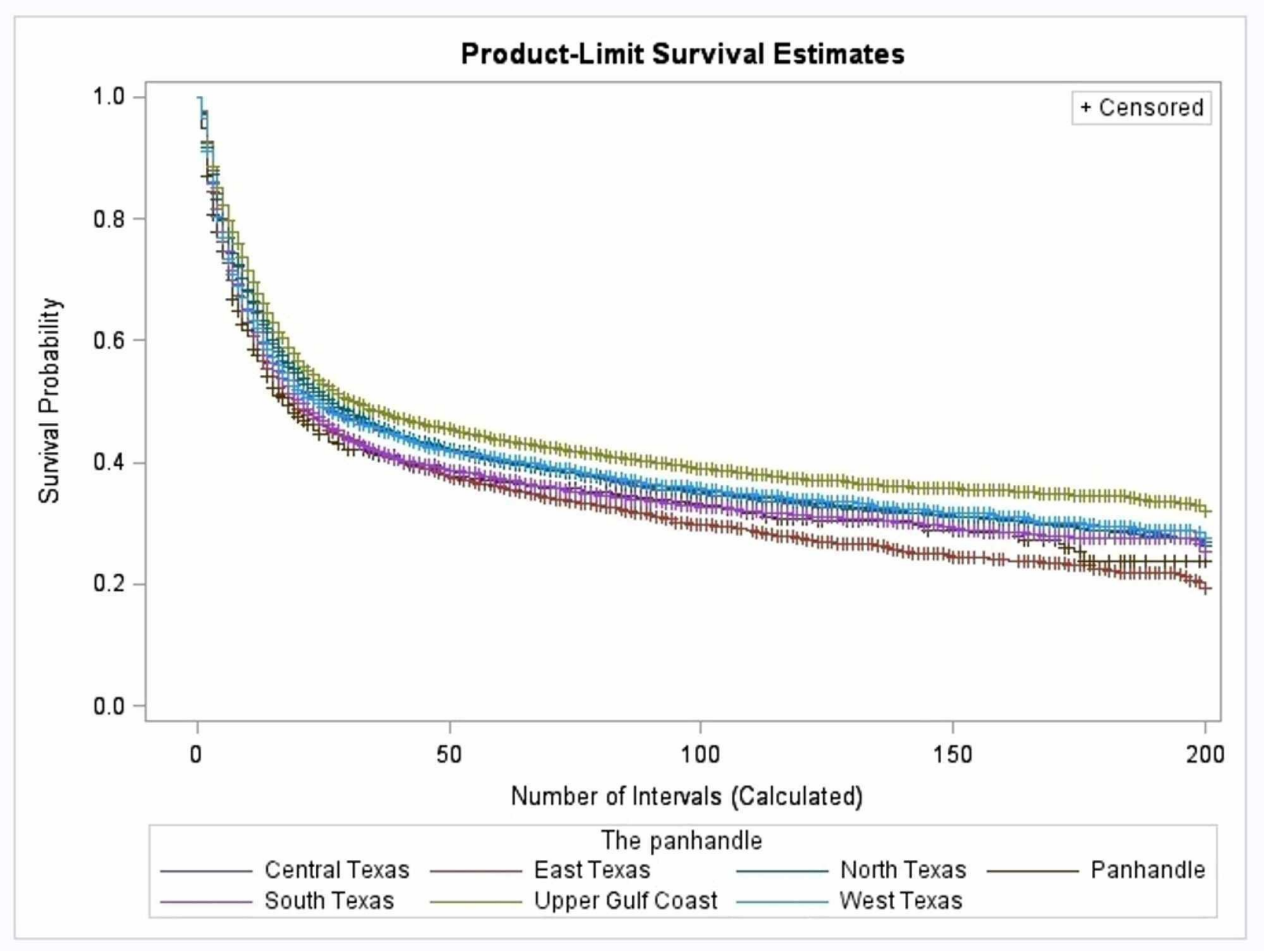
Kaplan Meier curve of survival probability by regions of Texas

The survival probabilities differ across the seven divided geographical regions of Texas (p-value < 0.0001). The highest median survival times were in the Upper Gulf Coast at 31 months (95% CI: 29-35%), North Texas at 27 months (95% CI: 25-29%), Central Texas at 25 months (95% CI: 22-28%), and West Texas at 24 months (95% CI: 20-28%). The lowest median survival times were seen in the Texas Panhandle at 18 months (95% CI: 14-23%), East Texas at 19 months (95% CI: 17-22%), and South Texas at 21 months (95% CI: 19-23%; Figure 1 and Table 1).

**Table 1:** Texas demographics by region

	Central Texas (N=3080)	East Texas (N=2523)	NA (N=619)	North Texas (N=7364)	Panhandle (N=541)	South Texas (N=3991)	Upper Gulf Coast (N=6123)	West Texas (N=2448)	Total (N=26689)	p-Value
Age at diagnosis										< 0.001
Mean (SD)	48.376 (23.758)	53.415 (22.754)	50.580 (24.321)	47.322 (23.352)	50.214 (24.410)	48.976 (24.802)	46.472 (23.280)	49.205 (24.860)	48.379 (23.813)	
Range	0.000 - 101.000	0.000 - 97.000	0.000 - 97.000	0.000 - 99.000	0.000 - 97.000	0.000 - 97.000	0.000 - 112.000	0.000 - 96.000	0.000 - 112.000	
Sex										0.194
Female	1366 (44.4%)	1159 (45.9%)	262 (42.3%)	3287 (44.6%)	266 (49.2%)	1805 (45.2%)	2718 (44.4%)	1132 (46.2%)	11995 (44.9%)	
Male	1714 (55.6%)	1364 (54.1%)	357 (57.7%)	4077 (55.4%)	275 (50.8%)	2186 (54.8%)	3405 (55.6%)	1316 (53.8%)	14694 (55.1%)	
Race										< 0.001
White	2796 (90.8%)	2232 (88.5%)	593 (95.8%)	6517 (88.5%)	514 (95.0%)	3862 (96.8%)	5033 (82.2%)	2362 (96.5%)	23909 (89.6%)	
Black	205 (6.7%)	261 (10.3%)	16 (2.6%)	618 (8.4%)	19 (3.5%)	89 (2.2%)	766 (12.5%)	64 (2.6%)	2038 (7.6%)	
Native	8 (0.3%)	5 (0.2%)	1 (0.2%)	20 (0.3%)	3 (0.6%)	5 (0.1%)	12 (0.2%)	5 (0.2%)	59 (0.2%)	
Asian	46 (1.5%)	9 (0.4%)	7 (1.1%)	174 (2.4%)	3 (0.6%)	17 (0.4%)	207 (3.4%)	6 (0.2%)	469 (1.8%)	
Other/unknown	25 (0.8%)	16 (0.6%)	2 (0.3%)	35 (0.5%)	2 (0.4%)	18 (0.5%)	105 (1.7%)	11 (0.4%)	214 (0.8%)	
Hispanic										< 0.001
No	2630 (85.4%)	2389 (94.7%)	334 (54.0%)	6413 (87.1%)	468 (86.5%)	1904 (47.7%)	4851 (79.2%)	1511 (61.7%)	20500 (76.8%)	
Yes	450 (14.6%)	134 (5.3%)	285 (46.0%)	951 (12.9%)	73 (13.5%)	2087 (52.3%)	1272 (20.8%)	937 (38.3%)	6189 (23.2%)	
Poverty level										< 0.001
0 - <5%	752 (24.4%)	102 (4.0%)	45 (7.3%)	2249 (30.5%)	87 (16.1%)	492 (12.3%)	1593 (26.0%)	174 (7.1%)	5494 (20.6%)	
5 - <10%	770 (25.0%)	449 (17.8%)	99 (16.0%)	2045 (27.8%)	144 (26.6%)	681 (17.1%)	1368 (22.3%)	389 (15.9%)	5945 (22.3%)	
10 - <20%	991 (32.2%)	1344 (53.3%)	229 (37.0%)	1928 (26.2%)	184 (34.0%)	986 (24.7%)	1784 (29.1%)	924 (37.7%)	8370 (31.4%)	
20 - 100%	558 (18.1%)	619 (24.5%)	239 (38.6%)	1113 (15.1%)	124 (22.9%)	1814 (45.5%)	1351 (22.1%)	955 (39.0%)	6773 (25.4%)	
Unknown	9 (0.3%)	9 (0.4%)	7 (1.1%)	29 (0.4%)	2 (0.4%)	18 (0.5%)	27 (0.4%)	6 (0.2%)	107 (0.4%)	

Survival by poverty index

**Figure 2:**
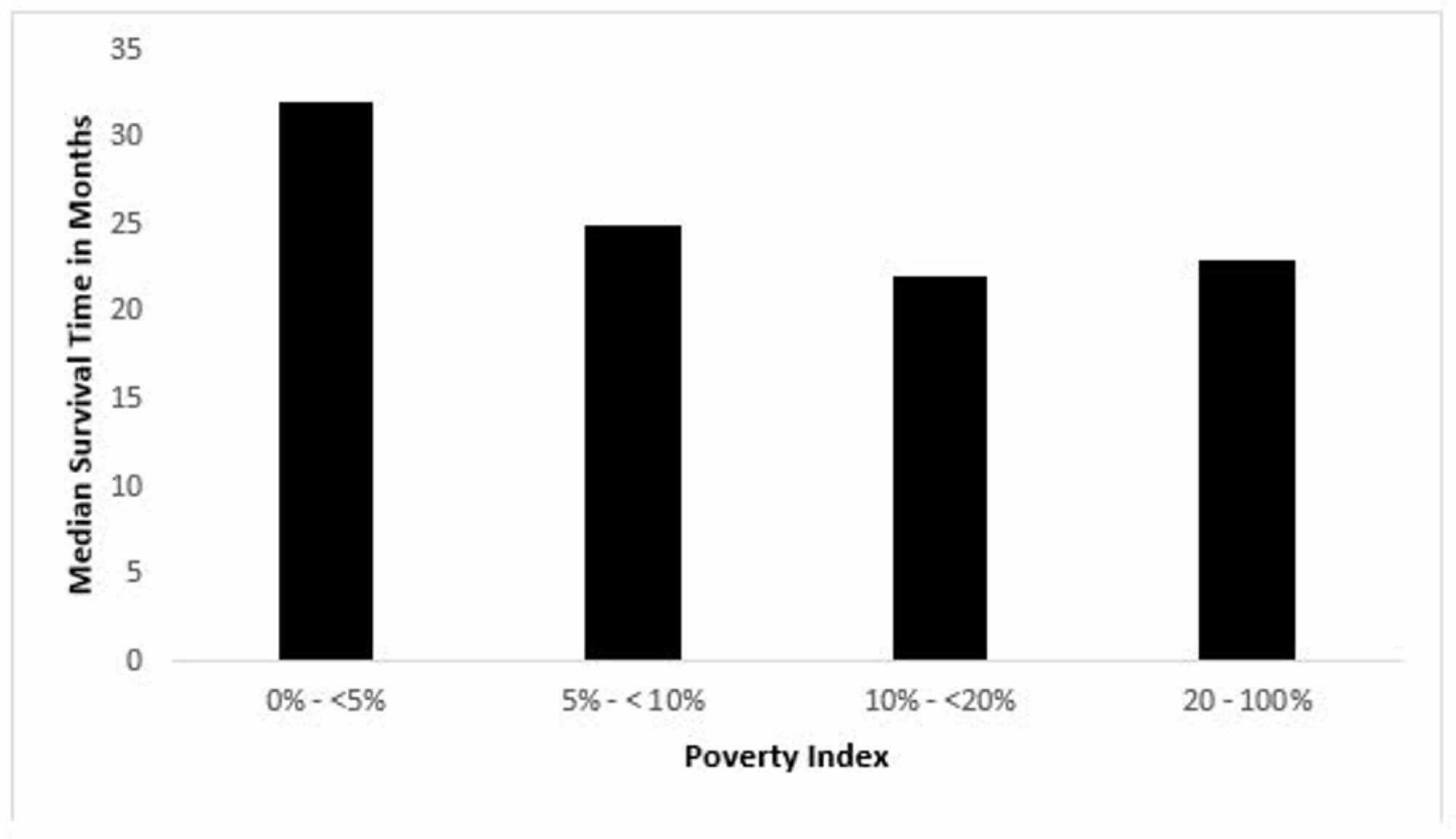
Median survival time versus poverty index

The survival probabilities differ across the four divisions of the poverty index (p-value < 0.0001). The highest median survival time of 32 months (95% CI 29-35%) was in the lowest poverty index of 0-5%. The second and third highest median survival times of 25 months (95% CI 23-27%) and 23 months (95% CI 23-26%) were in the poverty level of 5 - <10% and 20 - 100%, respectively. The lowest median survival time of 22 months (95% CI: 21-24%) was in the poverty level of 10 - 20% (Figure 2).

Survival by race

**Figure 3:**
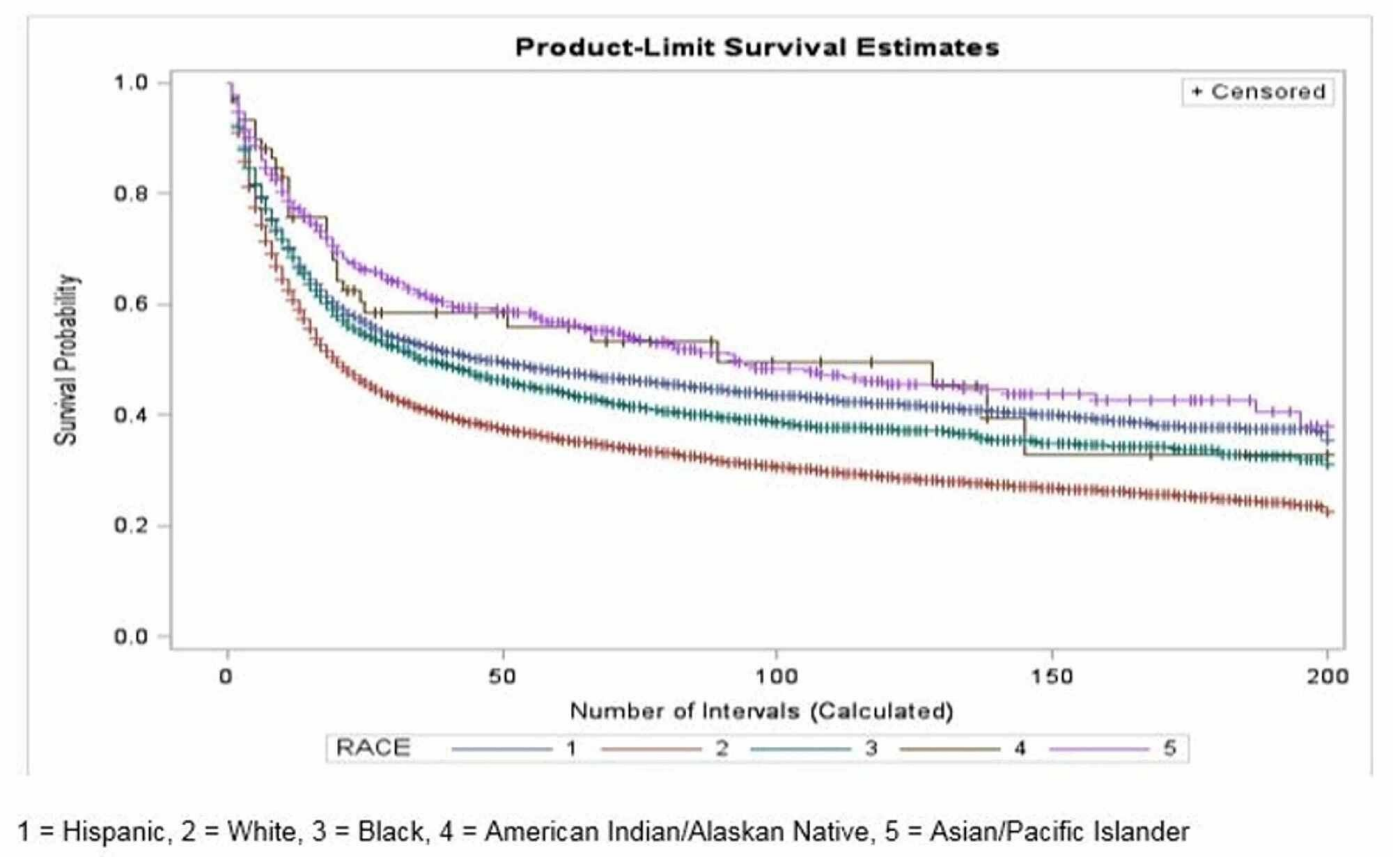
Kaplan Meier curve of survival probability by ethnicity

When stratified into five categories, Asians were found to have the highest median survival time of 92 months (95% CI: 72, 142). The category of American Indians and Alaska Natives patients had a median survival time of 89 months (95% CI: 21,138), then Hispanic patients at 47 months (95% CI: 41, 55), and Blacks at 36 months (95% CI: 31,45). Non-Hispanic Whites had the lowest median survival time of 20 months (95% CI: 19, 21). The difference in survival probabilities was significant (p-value <0.0001). Compared to Whites, Asians and Hispanics had hazard ratios of 0.70 (p-value < 0.0001) and 0.91 (p-value < 0.0001), respectively (Figure 3 and Table 2).

**Table 2:** Relative survival rates

Time	Male (n=14,694)	Female (n=11,995)	Hispanic (n=6,105)	White non-Hispanic (n=17,804)	Black (n=2,038)	American Indian/Alaskan native (n=59)	Asian/Pacific Islander (n=469)
1 month	97.4%	97.0%	97.28%	97.16%	97.01%	96.69%	98.08%
6 months	77.0%	75.30%	79.44%	74.26%	79.10%	88.14%	86.23%
12 months	64.3%	63.10%	68.56%	60.78%	68.55%	75.70%	77.40%
60 months	39.0%	41.20%	47.90%	35.80%	44.3%	55.97%	56.70%

Survival by gender

The relative survival rates differed across sex, with females having higher long-term survival rates at 60 months when compared to males. Males had a slight advantage in the first year after diagnosis, but females gradually gained an advantage when observed longitudinally at 5, 10, and 15 years. Overall, the hazard ratio of female to male was 0.872, corresponding to a 15% decrease in risk of death (p-value < 0.0001; Table 2).

## Discussion

Disparities in healthcare have historically been associated with minority groups, including Asians, African Americans, Hispanics, and American Indians/Alaska Natives. The role of such disparities has made a significant difference in prognoses of primary malignant brain tumor cases in the United States. Certain studies have shown that the survival of patients diagnosed with malignant brain tumors was increased 2-fold when the patient was insured and had established care with a primary care provider versus the uninsured without a primary care provider [3,4]. Given the lower rates of health insurance coverage among minority groups as compared to whites, it would be reasonable to conclude that minorities would have a survival disadvantage when diagnosed with a primary malignant brain tumor [8]. However, our study has shown that minorities have an advantage over whites in survival from primary malignant brain tumors. In fact, our findings revealed white patients diagnosed with a primary malignant brain tumor had an increased risk of death by 43% and 9.8%, respectively, when compared to Asian and Hispanic patients during our study period from 1995 to 2013. Additionally, our study revealed patients with a poverty index of 10 - 20% were found to have a higher chance of survival when compared to those with a poverty index exceeding 20% (Figure 2). This argues against the widespread belief that a higher poverty index is always associated with decreased survival.

Small genetic variations among the different races may contribute to the observed survival discrepancies as explained by recent discoveries of germ-line and gene-gene interactions in brain cancers [3,9,10]. Additionally, there have been multiple studies that investigated sex-specific hormone dependence of brain tumors that could account for the male-to-female incidence and survival differences, as also observed in our study [11-15]. The expanding evidence of genetic influence in incidence and survival of primary brain tumors may help explain not only the difference in prognoses by race and associated socioeconomic statuses but also by geographical region.

When investigating the differences in survival by region, our population samples were gathered from Texas only. As shown in the US Census Bureau, the demographics in selected regions and major metropolitan areas of Texas correspond to those of the United States, making Texas an ideal state to sample for this study [16]. Another study that looked at expansive regions of the United States reported statistically significant differences in patient survival of primary brain cancer among those categorized regions [17]. Similarly, our study has found statistical differences in survival from primary brain tumors by geographical region in Texas, with the highest median survival times in the Upper Gulf Coast, North Texas, and Central Texas, while the Texas Panhandle had the lowest median survival time. This could be explained in part by the fact that the Panhandle region has one of the highest incidence rates of primary brain tumors, and the Upper Gulf region has one of the lowest incidence rates in Texas [18]. Additionally, the genetic clustering that is present throughout the regions of Texas may also explain our study’s findings. Further investigations of the Texas demographics show that the majority of the population in South and West Texas is made up of Hispanics while those of the Upper Gulf Coast and East Texas are Blacks [12].

Another contributing factor to the survival differences observed by the region in Texas could be attributed to environmental conditions including heavy metal exposures, namely lead and iron, as well as petroleum and gas. Many studies have shown associations with these hazardous exposures in the environment with increasing incidences of brain tumors [18-21]. As Texas is a leading producer of oil in the United States, with petroleum and gas wells concentrated in varying regions of the state, incidence and survival may be affected accordingly [22]. From 2011 to 2014, the average annual age-adjusted incidence rate for primary CNS tumors (malignant and nonmalignant) in Texas was 24.53 per 100,000 population, which is slightly higher than the national average of 23.03 per 100,000 population as seen in the 2018 CBTRUS report [23]. As mentioned above, the disparities in healthcare including the number of primary care providers and access to health insurance also vary by region, thereby limiting the influence of geographical region alone.

This study focused on several variables that may play a longitudinal role in patient survival of primary brain cancers. A sharp fall in survival in the first two years after diagnosis of a primary brain tumor is seen across all variables, emphasizing the high mortality rate in the prognosis of primary brain cancer in general, regardless of sex, race, socioeconomic status, or geographical region. It is clear that no specific factor significantly influences survival in the early stages, which brings attention to the idea that improving healthcare for brain cancer patients should focus on areas of long-term modifications. In addition, due to these variables being interconnected with one another, as seen in the discussion of race, disparities in healthcare, and geographical location, it is difficult to make concrete conclusions on whether such factors are direct or indirect influences to survival.

Although we demonstrated a survival advantage in minorities with malignant brain tumors, it should be noted that this was a retrospective analysis studying malignant brain tumors as a collective whole instead of specific brain tumor by histological type. Furthermore, at the time of analysis, only datasets from 1995 to 2013 were available from the Texas Cancer Registry. Although no data after 2013 were analyzed, a meaningful trend from 1995 to 2013 can be appreciated to determine if differences among ethnic groups were present. The datasets provided from the Texas Cancer Registry were an average from 1995 to 2013, and it should be noted that population and diversity by geographic region of Texas may have changed in this time period. In future studies, subgroup analysis that correlates geographical region with ethnic group and tumor type should be performed, along with collecting additional criteria such as per capita income, average housing values, and the number of regional medical centers in each region to more accurately determine patient demographics and ease of access to medical care.

## Conclusions

Our study has demonstrated that selected minority groups, high socioeconomic status, and certain geographical locations were associated with lower risk of death from primary brain tumors. Race, age, sex, geographic location, and poverty index significantly predict the hazard of death reflecting strong genetic and environmental influence. As we attribute the underlying explanation to genetics and racial clustering by region, it may be important to investigate any direct links between patients of categorized race or region and gene-gene interactions in brain cancers. Any findings from studies that focus on the genetic make-up of patient populations will allow us to have a better understanding of primary brain cancer survival and how healthcare providers can appropriately modify their delivery of care.
